# Clinical Supervisors' and (Junior) Doctors' Experiences of Breastfeeding Risk Assessment, and Where We Go From Here

**DOI:** 10.1192/bjo.2024.373

**Published:** 2024-08-01

**Authors:** Erin Gourley, Megan Parsons

**Affiliations:** 1 Coventry and Warwickshire Partnership Trust, Coventry, United Kingdom; 2 Birmingham and Solihull Mental Health Trust, Birmingham, United Kingdom

## Abstract

**Aims:**

With recruitment and retention of NHS doctors an increasingly topical issue, the facilitation of a supported Return To Work (RTW) following a period of leave is particularly important. That the support provided takes a holistic approach to the wellbeing of the individual and their family unit is necessary if it is to be of the greatest success, especially with regards to new parents. The World Health Organization recommends breastfeeding until 2 years of age but in the UK just 0.5% of parents are breastfeeding at 1 year and, perhaps more significantly, 90% of breastfeeding parents stop before they would like to. Under the 2010 Equality Act, breastfeeding is a protected characteristic and legally, upon RTW, a breastfeeding parent must have a Breast-Feeding Risk Assessment (BFRA). The Health and Safety Executive have set out factors to be considered when completing BFRAs which enable the identification, mitigation or removal of risks that threaten breastfeeding, often via impacting the physical and mental health of the parent and child.

Our aim was to explore the experiences of both JDs and clinical supervisors in accessing and completing BFRAs in order to identify whether further work was required on this subject.

**Methods:**

A survey was sent to psychiatry JDs across the West Midlands inviting those who had RTW whilst breastfeeding to share their experiences. Another survey was sent to leads and supervisors across the region, exploring their confidence with BFRAs and their recommendations.

**Results:**

20 JDs responded. 16% received a BFRA with 5% being undertaken prior to RTW (best practise). For most of those who received one, it was a positive experience and 81% of those who did not receive one reported that they would have liked to but were either unaware that they existed or that they apply to children over 1 year.

36 consultants responded. 31% were aware of BFRAs with 9% feeling confident in completing one and none having had any training to do so. There was a strong sense that BFRAs should have a multi-disciplinary approach which contrasted with what occurred in reality.

**Conclusion:**

Identifying a lack of knowledge, as well as doctors’ need and desires regarding BFRAs, has resulted in a multifactorial approach to raising awareness of their existence, content and potential impact. Sessions for JDs and supervisors have been organised regionally and locally and there has been engagement with each trust in order to create a more uniform breastfeeding policy.

